# Contrast‐Enhanced Computed Tomography for Structural and Functional Evaluation of Ligament Microdamage

**DOI:** 10.1002/jor.70138

**Published:** 2026-01-28

**Authors:** Afifah H. Tsurayya, Jiri Jäntti, Petri Paakkari, Milka Poimala, Brian D. Snyder, Mark W. Grinstaff, Miitu K. M. Honkanen, Heta Mertano, Aapo Ristaniemi, Janne T. A. Mäkelä

**Affiliations:** ^1^ Department of Technical Physics University of Eastern Finland Kuopio Finland; ^2^ Diagnostic Imaging Center, Kuopio University Hospital Wellbeing Services Country of North Savo Kuopio Finland; ^3^ Boston Children's Hospital Boston Massachusetts USA; ^4^ Boston University Boston Massachusetts USA

**Keywords:** cationic nanoparticle, ligament, micro‐CT, neutral iodixanol

## Abstract

Detecting microstructural damage in ligaments remains a challenging case when no visible tearing occurs. This study introduces a novel application of micro‐CT for ligament evaluation in wet tissue state with potential for clinical translation. We utilize neutral iodixanol and cationic tantalum oxide nanoparticles (Ta₂O₅‐cNPs) for quantitative and qualitative imaging of microdamaged bovine ligaments. We hypothesize that neutral iodixanol reflects the ligament's functional alterations, while Ta₂O₅‐cNPs will depict ligament structure by highlighting biphasic differences between the interfascicular matrix and fascicular regions. To examine these hypotheses, bovine anterior (*N* = 6) and posterior (*N* = 5) cruciate ligaments were assigned to control and damaged groups. All samples underwent biomechanical tensile testing to quantify the functional properties. Ligaments in the damaged group were strained up to 16% to create microdamage. Young's modulus was significantly reduced by 68% in the damaged group relative to the healthy group (*p* < 0.001). Samples were then separately immersed in each contrast agent and imaged using micro‐CT at several timepoints during diffusion. The neutral iodixanol diffused faster compared to Ta₂O₅‐cNPs, while Ta₂O₅‐cNPs achieved approximately three times higher maximum partition. Although statistically significant differences in contrast agent partition between control and damaged groups were not observed, the findings demonstrate that (1) *P*_max_ of neutral iodixanol showed correlation with biomechanical properties specifically phase shift at frequencies of 0.1, 0.5, and 2 Hz indicating sensitivity to viscoelastic changes of the tissues, and (2) Ta₂O₅‐cNPs enable visualization of the ligament's structures, supporting their potential for three‐dimensional histological assessment.

## 1 Introduction

Ligaments are bands of tissue that connect bones with other bones, enabling joints' stability and mobility [1]. They consist of water, collagen, elastin, and proteoglycans and are organized hierarchically, similar to tendons. At the mesoscale, the ligament tissue divides into collagen‐rich fascicles (diameter 50 to 500 µm), which are separated by the interfascicular matrix (IFM) [2, 3, 4]. It is known that the interfascicular matrix and the fascicles have distinct material phases, such as richer collagen within the fascicle [5] and more abundant elastic fibers inside the IFM [2]. The presence of elastic fibers helps the tissue return to its original shape after significant deformation, while collagen contributes to its stiffness and strength [6].

Minor overloads, such as sprains, may cause microstructural damage to the ligament tissue, even though catastrophic failure or visual tears do not occur. Over time, these deteriorative changes may contribute to joint instability, pain, and degenerative conditions such as osteoarthritis [7]. However, current clinical imaging methods, such as magnetic resonance imaging, can typically detect ligament injuries only once the tissue shows macroscopically visible changes, such as partial or complete tears or swelling, making early microdamage difficult to diagnose [8, 9, 10]. A more sensitive imaging method is needed to spot the microscopic level of ligament tissue degradation and reduce the risk of further joint deterioration.

We propose that quantitative contrast‐enhanced computed tomography (CECT) could be used to detect the degradation of ligament tissues. CECT is a method to increase the X‐ray attenuation in the soft tissue to improve image contrast by utilizing high atomic number elements, such as iodine [11]. CECT has been successfully equipped to reveal the integrity, thickness, lesions, and macromolecule content in joint tissues, such as cartilage and meniscus, employing, for instance, cationic iodine‐based agents (CA2+ and CA4 +) to target negatively charged proteoglycans, and neutral gadoteridol to reflect the porosity of the tissues [12, 13, 14]. In recent years, the use of nanoparticles as contrast agents has gained attention, offering new possibilities for soft tissue characterization [15, 16, 17, 18]. Honkanen et al. used 0.2 µm bismuth nanoparticles to enable segmentation and detect lesions in cartilage, while Jäntti et al. demonstrated that cationic tantalum oxide nanoparticles (Ta₂O₅‐cNP) are sensitive to cartilage collagen content and orientation [15, 16]. Notably, due to the dense collagen network in cartilage, the small size of the nanoparticles is essential to ensure effective diffusion. Their cationic charge also enhances diffusion due to interactions with negatively charged proteoglycans, enabling more accurate mapping of tissue health [16]. Given the compositional similarities between cartilage and ligament, it suggests that these nanoparticles may also be suitable for imaging and assessing ligament tissues. This study represents, to our knowledge, the first use of quantitative CECT, specifically micro‐CT to detect structural and functional properties of bovine ligaments, in a wet‐tissue state (*i.e*., not chemically fixed). We subject the ligament samples to tensile testing to induce microdamage that alters their mechanical properties and then compare the altered properties to those of intact control samples. Following this, we conduct CECT on the damaged and control samples using two different contrast agents: commercially available neutral iodinated iodixanol and Ta₂O₅‐cNP synthesized in‐house. We hypothesize that the smaller‐sized contrast agent, neutral iodixanol, reflects tissue porosity [12], while the larger Ta₂O₅‐cNPs visualize ligament structure by highlighting biphasic variations between the IFM and fascicular regions of the ligaments. These hypotheses are based on the nanoparticles' positive charge and larger size, which are expected to promote accumulation in the negatively charged and more porous IFM [5, 19, 20].

## 2 Methods

### 2.1 Sample Preparation

**Figure 1:**
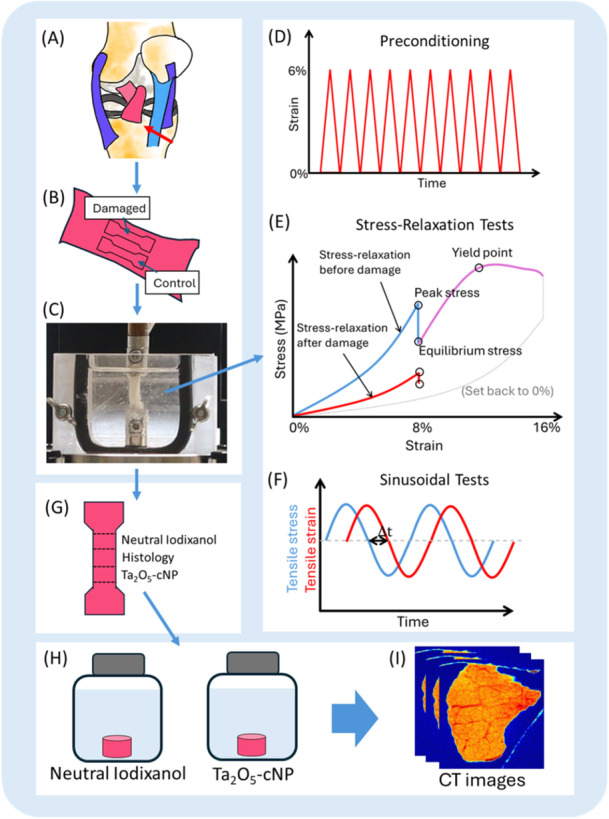
Workflow of the study. (A) Anterior and posterior cruciate ligaments (ACL and PCL, respectively) were harvested from bovine knees, and then (B) two samples were cut along the fascicle direction: one for control and one for damage. (C) Samples were mounted on a tensile testing device. (D) Preconditioning cycles were performed to stabilize the mechanical response. (E) A stress‐relaxation test was first applied at 8% strain (blue curve), and the damaged group samples were subsequently stretched up to 16% strain to induce microdamage (purple curve). After a 2‐second hold, the sample was returned to the original length (dashed line), and a second stress‐relaxation test was conducted (red curve). (F) Sinusoidal loading tests were performed before and after microdamage induction to assess viscoelastic properties. (G) Samples were then cut into three pieces for three analyzes: neutral iodixanol contrast‐enhanced computed tomography (CECT), Ta₂O₅‐cNP CECT, and histological reference. (H) The samples were immersed in the respective contrast agents; (I) Micro‐CT imaging was performed.

Anterior cruciate ligaments (ACL, *N* = 6) and posterior cruciate ligaments (PCL, *N* = 6) were dissected from bovine stifle joints (Figure 1A), kept in phosphate‐buffer saline solution (PBS) with protease inhibitors [5 mM, ethylenediamine‐tetra‐acetic acid disodium salt dihydrate (VWR International, Radnor, PA, USA) and 5 mM benzamidine hydrochloride hydrate (Sigma‐Aldrich Co., St. Louis, MO, USA)], and frozen at ‐23°C for 4‐8 weeks before experiments, a duration short enough to avoid storage‐related alterations in tissue mechanics [21]. The stifle joints were obtained from a local abattoir (Atria Suomi Oy); thus, ethical permission was not needed. Only cruciate ligaments and joints without visible abnormalities (e.g., discoloration or signs of damage) were included in the experiments. Before the experiments, the samples stored in vials were thawed in a lukewarm water bath. Two samples per ligament were cut (one for the control group and one for the damaged group) into dumbbell‐shaped tensile testing pieces using a punch tool with fascicle oriented along the axial direction (Figure 1B). The cut sample sizes varied in areas (from 7.0 to 16.5 mm^2^, average of 11.5 mm^2^) and initial length (from 15.2 to 23.8 mm, average of 19.6 mm). The width and thickness were measured using an optical microscope with a precision of 0.005 mm at the center, and the cross‐sectional area was calculated by assuming an elliptical shape. The sample length was defined as the clamp‐to‐clamp distance (precision of 0.1 µm).

### 2.2 Biomechanical Testing

Before the tensile testing measurements, double‐sided sandpapers (Mirox P80, Mirka Oy, Nykarleby, Finland) were attached to the end parts of the samples with glue (Loctite Precision, Henkel AG, Düsseldorf, Germany), then secured between the tensile‐testing grips (Figure 1C). During the measurement, the sample was immersed in PBS. Tensile testing was performed using a biomechanical testing system (Mach‐1 v500csst, Biomomentum Inc., Laval, QC, Canada), equipped with a 250 N (accuracy 12.5 mN) load cell. The measurement protocols are illustrated in Figure 1D–F. The initial length of the ligament sample (zero‐load length) was determined by applying a tensile stress of 0.05 MPa [22, 23, 24]. Samples were preconditioned with 10 loading‐unloading cycles to 6% strain with 2%/s strain rate, after which the initial length was redetermined [25, 26]. The preconditioning procedure was repeated five times to stabilize the mechanical behavior. The samples were then given 10 min of recovery before the tensile‐testing measurements. The tensile‐testing protocol consisted of one step of stress‐relaxation (8% strain with 8%/s and 10 min relaxation) and a sinusoidal test (0.5% strain amplitude at frequencies of 0.1, 0.5, 1, and 2 Hz). The damaged group samples were then stretched up to 16% to change the functional properties and structures with over‐physiological strain, and the stress‐relaxation testing at 8% strain and the sinusoidal testing were repeated to evaluate the altered biomechanical properties. The 16% strain level was selected after conducting preliminary investigations to identify the strain magnitude sufficient to reach the yield point without causing physical failure and visible damage. The details of the biomechanical data analyzes and equations can be found in Supporting Information S1.

### 2.3 Micro‐CT Imaging

#### 2.3.1 Sample and Contrast Agent Preparation

After the biomechanical measurements (Figure 1C), the samples were cut transversely into three pieces (Figure 1G) and frozen in PBS with inhibitors at −23°C until the micro‐CT measurements. A conventional contrast agent, neutral iodixanol (Visipaque^TM^ 320 mg∙I/mL, GE Healthcare, Oslo, Norway), was prepared with a 12 mg∙I/mL concentration and an osmolality of 308 mOsm/kg. The Ta_2_O_5_‐cNP was synthesized as previously described, with the coating step modified to use solely the quaternary ammonium cation ligands (*N‐*trimethoxysilylpropyl*‐N*,*N*,*N‐*trimethylammonium chloride), and the core of the particle size was 3.8 nm, measured using transmission electron microscopy [17]. The zeta potential (surface charge) was determined to be 45.3 ± 3.0 mV using electrophoretic light scattering (Zetasizer Nano ZS, Malvern Instruments Ltd., Malvern, UK). The Ta_2_O_5_‐cNP formulation was prepared at a concentration of 20 mg/mL, with a pH of 7.4, and an osmolality of 309 mOsm/kg. To minimize dilution effects, the contrast agent bath volume was set to 6.5 mL, corresponding to 100 times the average sample volume.

#### 2.3.2 Contrast‐Enhanced Computed Tomography

After thawing at room temperature, the ligament samples were mounted onto a Styrofoam holder to ensure stable positioning during imaging. Initial scans were performed using a micro‐CT scanner (Nikon XT H 225, Nikon Metrology Europe, Leuven, Belgium) to obtain native sample geometry data without contrast agents. Subsequently, the samples were immersed in the contrast agent baths (one subsample of each sample per contrast agent). CECT was conducted at several timepoints with the samples scanned in air: 0.75, 1.5, 3, 5, 8, and 22 h (iodixanol), and 3, 8, 22, 46, and 72 h (Ta_2_O_5_‐cNP). At each timepoint, samples were removed from the baths and placed back into the holder, stacked six per scan, to prevent movement during imaging. A water tube was imaged together with the samples for Hounsfield Unit (HU) conversion purposes. The CT images were obtained with a tube voltage of 100 kVp and 1.5 mm aluminum filter was applied. The samples were imaged using a continuous scan mode, acquiring 3600 projections at one frame per projection. The total duration of one scan was approximately 21 min. The projection data were reconstructed using Inspect‐X‐3D CT Reconstruction Software (Nikon Metrology NV) and the isotropic voxel size in the reconstruction was 16.99 μm ^3^.

#### 2.3.3 CT Image Analysis

##### 2.3.3.1 Quantitative Analysis

Reconstructed CT images were analyzed using custom‐made MATLAB code (R2022b, The MathWorks Inc., Natick, MA, USA). The samples were segmented from the background using MATLAB function “imsegkmeans3,” volume segmentation based on K‐means clustering (*k* = 2). After the volumetric segmentation, geometrical smoothing was applied in each 2‐dimensional slice by 10 pixels, shrinking the image using the “bwmorph”‐function, and removing small areas under 2000 pixels with the “bwareaopen”‐function. The maximum contrast agent partition, *P*_max_, representing the relative accumulation of the contrast agent in the tissue (i.e., its concentration normalized to the bath concentration) in the sample at diffusion equilibrium, was determined through exponential fitting relative to the solution partition:

$$\mathrm{Partition}\left(t\right)=P_{\max}\times [1-\text{exp}(-t/\tau )],$$

where $P_{\max}$ is equilibrium contrast agent partition, t is diffusion timepoint, and τ is diffusion time constant that describes the time required to reach 63.2% of the equilibrium partition [27]. Due to the large volume of the contrast agent bath relative to the sample size, dilution effects during diffusion were considered negligible based on the dilution effect error estimation.

##### 2.3.3.2 Qualitative Analysis

Two samples imaged with CT using Ta_2_O_5_‐cNP as a contrast agent were selected for qualitative assessment based on adequate structural contrast. Following background removal, fascicles were segmented from the sample using the MATLAB “kmeans” function with *k* = 3 to cluster pixel attenuation values in each image slice. The choice of “*k*” was made to minimize partial volume effects and to ensure effective differentiation between the fascicles and the interfascicular matrix. Based on the attenuation characteristics in this dataset, “Cluster 1” corresponded to the pixel group (region) with the lowest attenuation values, “Cluster 2” to intermediate values, and “Cluster 3” to the highest. The segmented fascicles were formed by subtracting Cluster 3 from the original image. Additionally, we manually selected regions of interest (ROI) to differentiate the IFM and fascicular regions in all of the samples (illustrative images are shown in the Supporting Information S2).

### 2.4 Histological Analysis

Histological analysis was performed on two tissue pieces derived from the same ligament bundles used in the qualitative CT assessment (Section 2.3.3.2). After fixation in 10% formalin for over 48 h, the sample was dehydrated with ethanol and embedded in paraffin. Each sample was halved and cut transversally into 5‐µm‐thick sections. Subsequently, the sections were stained with Verhoeff‐van Gieson to stain the collagen (red), elastin (black), cells (blue to black), and other parts (yellow). The images were acquired with a Zeiss Stemi 508 Stereo Microscope (Carl Zeiss Microscopy LLC, White Plains, NY, USA) with 1.25× magnification.

### 2.5 Statistical Analysis

Statistical analysis was performed in MATLAB. Biomechanical properties, contrast agent partition, and diffusion parameters were compared between control and damaged samples using the Wilcoxon signed‐rank test for dependent data. Spearman's rank correlation coefficient was utilized to evaluate the correlation between biomechanical parameters and contrast agent partition and diffusion parameters. Data from control and damaged groups were pooled for these correlations, except for the damage‐specific parameters, where only data from the damaged group were available and thus analyzed. The difference between the damaged and control groups and the correlation were considered statistically significant when *p* < 0.05.

## 3 Results

### 3.1 Biomechanical Testing

One PCL sample was excluded from the analysis due to a failed measurement, resulting in a final sample size of 6 ACLs and 5 PCLs. That sample was also omitted from the CECT analysis. Young's modulus was significantly lower in the damaged group compared to the control group (*p *< 0.01, Table 1). All the samples in the damaged group reached the yielding point (described as yield strain and yield stress), validating the micro‐structural alterations. Additionally, the level of damage was evaluated by damage parameters ($D_{\sigma },D_{\varepsilon },\lambda_{\varepsilon }$). Phase shift at 0.1 Hz in the damaged group decreased considerably (*p *< 0.05), whereas phase shift at the other frequencies did not significantly differ between the control and damaged groups.

**Table 1:** Biomechanical properties of healthy and damaged groups (mean ± standard deviation) determined using tensile and sinusoidal‐load testing protocols. Statistical differences between the groups are indicated with Wilcoxon signed‐rank test *p*‐values. “ns” stands for *p* > 0.05.

Symbol	Property	Unit	Control group	Damaged group	*p*
*E*	Young's Modulus	MPa	70.10 ± 26.67	22.13 ± 14.48	< 0.001
$\mathrm{Rati}o_{\mathrm{PE}}$	Peak‐equilibrium ratio	—	1.80 ± 0.32	1.44 ± 0.14	< 0.001
$A_{1}$	Amplitude in fast relaxation	MPa	0.88 ± 0.33	0.11 ± 0.07	< 0.001
$\theta_{1}$	Characteristic time in fast relaxation	s	2.56 ± 0.78	2.25 ± 0.60	ns
$A_{2}$	Amplitude in slow relaxation	MPa	0.49 ± 0.18	0.06 ± 0.04	< 0.001
$\theta_{2}$	Characteristic time in slow relaxation	s	137.18 ± 14.35	120.58 ± 24.04	< 0.05
γ	0.1 Hz phase shift	°	4.13 ± 0.63	3.55 ± 0.54	< 0.05
	0.5 Hz phase shift		3.27 ± 0.50	3.01 ± 0.55	ns
	1 Hz phase shift		3.08 ± 0.51	3.15 ± 0.57	ns
	2 Hz phase shift		2.95 ± 0.49	3.20 ± 0.98	ns
$D_{\sigma }$	Damage parameters	—	—	0.82 ± 0.11	N/A
$D_{\varepsilon }$		—	—	0.75 ± 0.05	N/A
$\lambda_{\varepsilon }$		—	—	0.04 ± 0.01	N/A
$\varepsilon_{\text{yield}}$	Yield strain	—	—	0.12 ± 0.01	N/A
$\sigma_{\text{yield}}$	Yield stress	MPa	—	5.54 ± 2.52	N/A

### 3.2 Contrast‐Enhanced Computed Tomography

**Figure 2:**
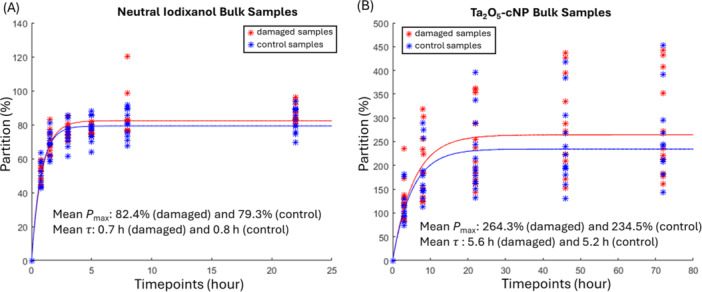
Bulk partition of contrast agents in ligaments (relative attenuation normalized to the attenuation of the contrast agent bath). (A) Electrically neutral iodixanol and (B) positively charged Ta_2_O_5_‐cNP, shown for damaged samples (red) and control samples (blue). Mean equilibrium partition ($P_{\max}$) and diffusion time constant (τ) are also presented for both groups. The y‐axis has been adjusted for each contrast agent. No significant differences in either $P_{\max}$ or τ were observed between the control and damaged groups for either contrast agent.

Neutral iodixanol and Ta_2_O_5_‐cNP contrast agents diffused efficiently into bovine ligament, reaching equilibrium partition ($P_{\max}$) of 81 ± 6% and 249 ± 90%, respectively (Figure 2). Iodixanol reached its equilibrium earlier compared to Ta_2_O_5_‐cNP (*p *< 0.001), seen as a lower diffusion time constant value (*τ*, describing the time to reach 63.2% of its equilibrium partition), for iodixanol (0.82 ± 0.20 h) than for Ta_2_O_5_‐cNP (5.38 ± 1.36 h). Both $P_{\max}$ and *τ* showed no differences between the control and damaged groups for either contrast agent.

**Figure 3:**
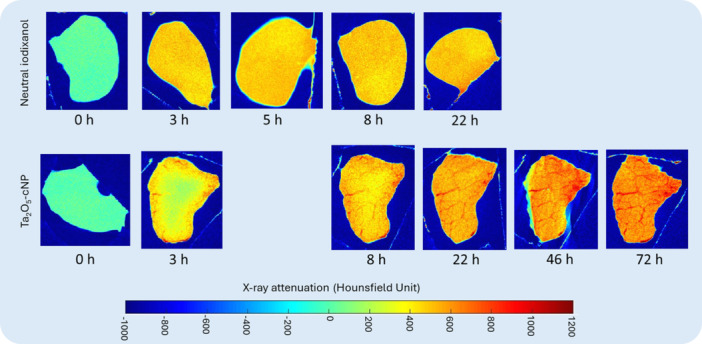
Two‐dimensional CT images (by averaging 10 consecutive slices = ~170 µm) of ligament samples immersed in neutral iodixanol (top row) and Ta_2_O_5_‐cNP (bottom row) acquired at each timepoint. Initial bath attenuations are shown on the right. The slices were selected from the central region of the sample. As the samples were not co‐registered between timepoints, the representative slice might not be from the exact same location at each timepoint.

The diffusion of both contrast agents into samples is further illustrated through changes in X‐ray attenuation in two representative samples in Figure 3. The attenuation maps visually support the quantitative findings in Figure 2, confirming that iodixanol reached its equilibrium state in the tissue more rapidly compared to Ta_2_O_5_‐cNP. Notably, attenuation values with Ta_2_O_5_‐cNP exceeded those of the contrast agent (indicating partition > 100%), while iodixanol remained below bath attenuation, mirroring the patterns reported in the bulk diffusion data.

**Figure 4:**
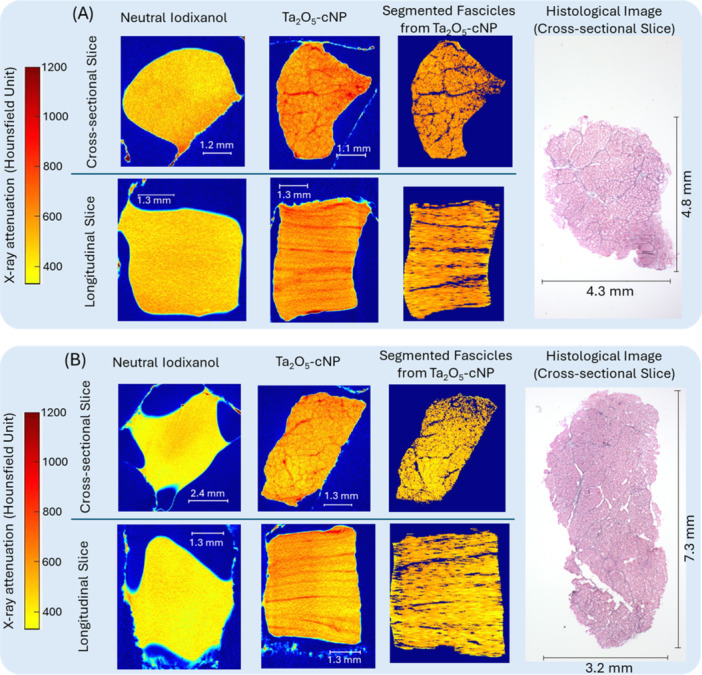
Two‐dimensional CT image slice of two ligament samples, (A) a sample from the damaged group, and (B) a sample from the control group. The original CT images of iodixanol and Ta_2_O_5_‐cNP and the segmented fascicles from Ta_2_O_5_‐cNP CECT are shown in cross‐sectional (z‐direction) and longitudinal (y‐direction) views. The CECT images (original images and segmented fascicles) were averaged over 10 slices (~170 µm) to reduce the noise for visualization purposes. The histological samples from the adjacent sides (see Figure 1G) are displayed in the cross‐sectional orientation for comparison.

The qualitative inspection of the diffusion of Ta_2_O_5_‐cNP into samples revealed local variation in attenuation. Representative two‐dimensional image slices of two samples (A and B) from cross‐sectional and longitudinal views are shown in Figure 4A,B. In sample A, when immersed in Ta_2_O_5_‐cNP, the average attenuation was 603 ± 22 HU in the fascicles and 795 ± 29 HU in the IFM, and in sample B, it was 519 ± 21 HU in the fascicles and 696 ± 22 HU in the IFM.

### 3.3 Correlation Analysis Between Biomechanics and CECT

**Figure 5:**
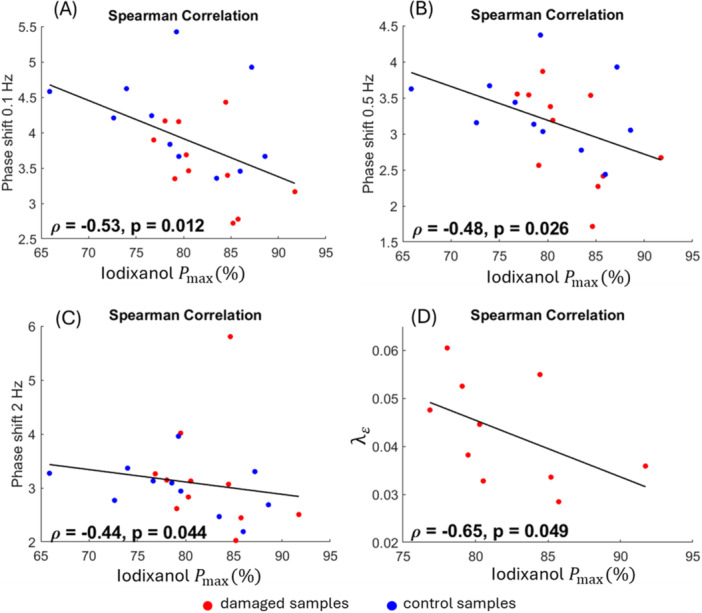
Spearman correlations between bulk neutral iodixanol $P_{\max}$ with (A) phase shift 0.1Hz, (B) phase shift 0.5 Hz, (C) phase shift 2 Hz, and (D) damage parameter $\lambda_{\varepsilon }$.

The $P_{\max}$ of neutral iodixanol showed negative correlations (*ρ *= −0.53 to −0.44, *p *≤ 0.05) with phase shifts at all frequencies except 1 Hz (Figure 5), with correlations determined using data pooled from both control and damaged groups. The $P_{\max}$ of neutral iodixanol also had a negative correlation with a parameter measuring the level of damage, $\lambda_{\varepsilon }$ (*ρ *= *−*0.65, *p *= 0.049), where the correlation was determined only with the damaged group. For the rest of the parameters described in Table 1, no correlation with $P_{\max}$ of iodixanol was found. The $P_{\max}$ of Ta_2_O_5_‐cNP did not show any correlation with biomechanical properties.

## 4 Discussion

Only a limited number of studies have been published on the utilization of CECT to visualize ligament or tendon structures, and those that do typically involve formalin‐fixed or chemically dried samples [28, 29, 30, 31]. In this study, we present the first application of CECT for assessing both structure and function in fresh (unfixed) bovine ligaments, aiming to assess its feasibility for future clinical translation.

Our findings demonstrate that both conventional molecular contrast agents (iodixanol) and novel nanoparticle‐based agents (Ta₂O₅‐cNP) provide complementary insights into ligament properties through CECT. The molecular iodixanol reached equilibrium faster than Ta₂O₅‐cNP (*p* < 0.001), with a lower diffusion time constant (τ = 0.82 ± 0.20 h), and a maximum partition of 81 ± 6%. As the first study to investigate the diffusion of neutral contrast agent into ligaments, the obtained results have been compared to prior cartilage studies. In bovine cartilage, neutral iodixanol reached a maximum partition of 62.1% (21 mM batch concentration, imaged by micro‐CT) [32]. Another study performed dual contrast agents (CA4+ and neutral gadoteridol) in human articular cartilage, and the maximum partition of the neutral gadoteridol (10 mg Gd/mL bath concentration) was 52%, imaged using a clinical CT [33]. Although cartilage has a higher water content (65%–85%) than ligament (60%–70%) [34, 35], and iodixanol is hydrophilic, the relative partition in ligament was higher or at least similar. This surprising result suggests that other factors, such as matrix density or microstructure, may play a more prominent role in regulating diffusion in ligaments than previously thought.

Iodixanol demonstrated potential for functional assessment of ligaments. Its maximum partition (*P*_max_) showed statistically significant negative correlations with viscoelastic properties—particularly phase shift values at 0.1, 0.5, and 2 Hz—as well as with a strain‐based damage parameter (*λ*_*ε*_). These results suggest that samples with higher iodixanol uptake exhibit lower energy dissipation and altered fluid‐dependent behavior. Notably, correlations with phase shift were most prominent at 8 h (across all tested frequencies; see Supporting Information Section S3), but were no longer observed at 22 h, indicating possible oversaturation. Other properties, such as Young's modulus and stress‐relaxation measures, did not correlate with iodixanol uptake, suggesting that iodixanol—a small, neutral, hydrophilic molecule—is more sensitive to viscous than elastic changes. Interestingly, its negative correlation with one of the damage parameters, *λ*_*ε*_, implies that severe strain may hinder diffusion, possibly due to altered matrix integrity. While no group‐wise differences in uptake were observed, these correlations highlight iodixanol's potential to reflect subtle, microstructural functional changes in ligament tissue. Further optimization, particularly to reduce diffusion time, could enhance its translational relevance.

Ta₂O₅‐cNP reached the equilibrium more slowly (τ = 5.38 ± 1.36 h) compared to the neutral iodixanol and a significantly higher maximum partition (249 ± 90%). From Figure 3, the nanoparticle penetration into the sample is visually apparent, particularly at the 3 h and 8 h timepoints. Although ligaments contain relatively low levels of proteoglycans (< 3% of dry weight) [36], they are relatively rich in elastin (0.25% to 10% of the tissue's dry weight) [2] and the IFM may exhibit a slightly negative charge due to its matrix composition. This negatively charged environment likely promotes the binding and retention of cationic nanoparticles. In an earlier study, using Ta_2_O_5_‐cNPs (bath concentration of 30 mg/mL, nanoparticle size of 2.55 nm) in healthy equine cartilage, the maximum partition was 800% in the medial femoral condyle and 372% for the distal intertrochlear groove [16]. Cartilage contains a significantly higher proteoglycan content (~15% of the wet weight) [37], resulting in a greater fixed‐charge density. These findings suggest that while proteoglycan content is a major determinant of positively charged nanoparticle uptake in cartilage, other negatively charged matrix components, such as elastin, may play a critical role in facilitating cationic nanoparticle diffusion into ligaments.

Using Ta_2_O_5_‐cNP as a contrast agent successfully revealed distinct regional differences in ligament attenuation, with patterns that correspond closely to the structural division between the IFM and fascicles, as confirmed by the microscopic images (Figure 4). In contrast, neutral iodixanol produced a homogeneous attenuation profile, showing no visible structural differentiation. The IFM is known to contain more elastin and proteoglycans and to have a less dense collagen network than fascicular regions [5, 19, 20]. Our results are in strong agreement with these structural properties, as the cationic nanoparticles were more concentrated in the IFM. However, contrast distribution across the ligament was not entirely uniform in all samples, which may reflect underlying biological variability or unbound nanoparticle presence. Due to the lack of consistent clustering‐based segmentation in many of the samples, manually segmented regions of interest were analyzed to differentiate the IFM and fascicular regions. Although this approach allowed structural analysis, it introduced subjectivity and may have limited sensitivity to more subtle or spatially complex variations in ligament architecture.

We observed considerable variability in Ta₂O₅‐cNP partitions across ligament samples, with mean attenuation values of 864 ± 311 HU in fascicles and 1088 ± 349 HU in IFM. This variability may reflect natural differences in tissue composition but could also result from non‐specific accumulation of unbound nanoparticles. Further optimization, such as post‐incubation washing, may improve image contrast and reproducibility. Previous studies support this approach: for example, a study using 15 mg∙I/mL concentration of CA4+ in bovine cartilage (24 h immersion) showed that a 48 h washout period led to partial removal, reducing the original 25 mg∙I/mL concentration to 13 mg∙I/mL within the tissue, indicating that a significant fraction was not permanently bound [38]. Similarly, another study reported a 50% reduction in CA4+ concentration after 24 h of saline immersion [39, 40]. These findings suggest that washing protocols, as well as diffusion time and nanoparticle concentration, may be key to enhancing reproducibility and image clarity in future applications.

To confirm that the samples in the damaged group exhibited functional changes, we first quantified their biomechanical properties before imaging. The significant decrease in Young's modulus in the damaged group verified a considerable reduction in ligament stiffness. Additional parameters, including yielding points and the damage parameters ($D_{\sigma }$, $D_{\varepsilon }$, and $\lambda_{\varepsilon }$), further confirmed functional alterations. In the current study, the mean damage parameter $D_{\sigma }$ was 0.82, indicating a significant decrease in the maximum stress after damage. In a previous study by Buckley et al., the damage parameter $D_{\sigma }$ was reported to be 0.61–0.69 and 0.78–0.85 in aged and injured mouse patellar tendons, respectively [41]. This suggests that the degree of microdamage induced here falls within a biologically relevant range for injury modeling, even if direct comparison is limited due to species and tissue differences. The yield strain in the current study ranged from 0.11 to 0.14. In comparison, Ristaniemi et al. reported slightly higher values for $\varepsilon_{\text{yield}}$ of bovine ligaments (0.19 and 0.18 for ACL and PCL, respectively). Furthermore, $\sigma_{\text{yield}}$ in this study ranged from 1.35 MPa to 9.89 MPa, which is substantially lower than previously reported values for bovine ligaments*—*30 MPa for ACL and 28 MPa for PCL [23]. One possible factor affecting these discrepancies is the longer sample length in our study (~19.6 mm vs ~10 mm). As Legerlotz et al. demonstrated, strain to failure and stress to failure significantly decrease with increasing specimen length of the bovine fascicle [42]. The viscoelastic response, particularly the phase shift at 0.1 Hz, was also significantly altered in damaged tissues. This finding aligns with Buckley et al. [43], who showed that lower frequencies better capture fluid flow‐related behaviors like fascicle sliding, a key mechanism of ligament viscoelasticity [44]. Finally, the observed changes in the slow relaxation ($\theta_{2})$ between groups provides additional support for functional alterations [45]. Taken together, these results suggest that the induced damage was sufficient to create detectable and biologically meaningful impairments in ligament mechanics, consistent with early‐stage degeneration or subclinical injury.

Although biomechanical testing confirmed functional impairment in the damaged group, no significant differences in contrast agent partition were observed between the control and damaged groups. This suggests that the current CECT setup may not yet be sensitive enough to capture microdamage‐related changes. The lack of full 3D segmentation between IFM and fascicles hindered the assessment of whether localized increases in IFM volume or region‐specific differences in contrast agent diffusion occurred following damage. Improving nanoparticle design, such as tuning particle size or surface charge, and adjusting the diffusion protocol, could improve detection sensitivity. Pre‐scan rinsing may help improve quantification by removing unbound particles in experimental settings, though such procedures may not be directly applicable in clinical workflows.

There were several limitations in the present study. First, the relatively low number of samples (*N *= 11) reduced the statistical power, particularly for the quantitative CECT, though it was sufficient to detect biomechanical changes; thus, the observed results provide valuable information. Second, fresh, unfixed samples were difficult to secure consistently in the sample holder during micro‐CT imaging, preventing accurate co‐registration. Albeit, this limitation did not affect the quantitative CECT, because the analyzes depend only on the bulk attenuation, regardless of image geometry. Third, the lack of structural/biochemical assessment limited us to quantify, for example, the amount of elastin and proteoglycan that might be correlated with the concentration of the cationic nanoparticles. However, this correlation between fixed charge density and Ta_2_O_5_‐cNP partition has been shown earlier for cartilage [13], and we expect it to hold for ligaments. Fourth, the same scan settings were used for both contrast agents during CECT imaging. While this approach ensured consistency, further optimization, for example, tube voltage, exposure time, and reconstruction settings, could improve image quality for each agent. Finally, the open‐ended geometry of the ligament samples likely allowed diffusion through the cut ends, potentially overestimating contrast agent accessibility compared to in vivo conditions.

To conclude, this study explores the application of micro‐CT‐based CECT using both neutral iodixanol and a custom‐developed cationic nanoparticle (Ta₂O₅‐cNP) to assess the structure and function of fresh bovine ligaments. Despite some limitations, our results demonstrate that tailored contrast agents can reflect structural features and correlate with tissue mechanical properties, reinforcing the method's potential for non‐destructive soft tissue evaluation. Neutral iodixanol reflected viscoelastic properties through its partition behavior, although it did not statistically differentiate the micro‐damaged group from controls. Still, its correlation with phase shift parameters suggests sensitivity to subtle, early‐stage functional changes. Ta₂O₅‐cNP effectively visualized ligament microstructure, especially fascicular organization, highlighting its utility for 3D histological imaging. While the approach is promising, clinical translation requires improved delivery methods for intra‐articular ligaments, minimizing physiological clearance effects, and optimizing imaging parameters for each contrast agent. Nevertheless, this study provides a foundational step toward the development of tailored imaging protocols that can differentiate between structural and functional features in ligaments, contributing to the broader advancement of high‐resolution soft tissue imaging using contrast‐enhanced CT.

## Author Contributions

Afifah H. Tsurayya contributed to research design, CT, histological and biomechanical data acquisition, data analysis and interpretation, article drafting and revision. Jiri Jäntti and Petri Paakkari participated in research design, CT image acquisition, data interpretation, and article revision. Milka Poimala synthesized and characterized the nanoparticles and revised the article. Brian D. Snyder, Mark W. Grinstaff, and Miitu K. M. Honkanen were involved in research design and article revision. Heta Mertano contributed to research design, CT data analysis and interpretation, and article revision. Aapo Ristaniemi participated in research design, histological image acquisition, biomechanical data analysis and interpretation, and article revision. Janne T. A. Mäkelä contributed to research design, data analysis and interpretation, and led article drafting and revision. All authors reviewed and approved the final submitted article.

## Supporting information

Supplementary material_revised.pdf.
