# Supplementary material for: Contrast‐Enhanced Computed Tomography for Structural and Functional Evaluation of Ligament Microdamage
Source: J Orthop Res. 2026 Jan 28;44(2):e70138. doi: 10.1002/jor.70138 (PMC12852979; doi:10.1002/jor.70138)
Supplement: Supplementary file 1 — Supplementary material_revised.pdf. [file JOR-44-0-s001.pdf]

Supplementary material

CONTRAST-ENHANCED COMPUTED TOMOGRAPHY FOR STRUCTURAL AND FUNCTIONAL EVALUATION OF LIGAMENT MICRODAMAGE

Afifah H. Tsurayya[1,2], Jiri Jäntti[1,2], Petri Paakkari[1,2], Milka Poimala[1,2], Brian D. Snyder[3], Mark W. Grinstaff[4], Miitu K.M. Honkanen[2], Heta Mertano[1,2], Aapo Ristaniemi[1,2], Janne T.A. Mäkelä[1]

[1]*Department of Technical Physics, University of Eastern Finland, Kuopio, Finland*

[2]*Diagnostic Imaging Center, Kuopio University Hospital, Kuopio, Finland*

[3]*Boston Children's Hospital, Boston, MA, United States*

[4]*Boston University, Boston, MA, United States*

Corresponding author:

Afifah Tsurayya, M.Sc.

Department of Technical Physics

University of Eastern Finland

POB 1627, FI-70211 Kuopio

Finland

Tel. +358 50 577 6486

E-mail: afifah.tsurayya@uef.fi

Running title: Computed tomography for ligament

S1) Biomechanical data analysis

The biomechanical measurement data were analyzed using custom-made MATLAB code (R2022b, The MathWorks, Inc., Natick, MA, USA). Stress (MPa) was defined as $\sigma = \frac{F}{A_0}$, where F is measured force and A_0 is the initial cross-sectional area, and strain (%) was defined as $\varepsilon = \frac{L-L_0}{L_0}$, where L is the current length and L_0 is the initial length (zero-load length). The peak stress and the equilibrium stress were determined for each relaxation test, and the ratio between peak and equilibrium is:

$$Ratio_{PE} = \frac{\sigma_{\text{peak}}}{\sigma_{\text{equilibrium}}}. \quad (1)$$

Young's modulus was determined as the maximum slope found in the stress-strain curve, corresponding to the linear region, by evaluating the slope at each point with a 0.8% strain interval. Stress-time data during the relaxation phase were fitted with a bi-exponential function[1,2] to calculate the relaxation rate as follows:

$$\sigma(t) = \sigma_{\text{peak}} - Y_1\left(1 - e^{-\frac{t}{\theta_1}}\right) - Y_2\left(1 - e^{-\frac{t}{\theta_2}}\right), \quad (2)$$

where $\sigma(t)$ is stress as a function of time, Y_1 is the fast relaxation amplitude, θ_1 is characteristic time in fast relaxation, Y_2 is the slow relaxation amplitude, and θ_2 is characteristic time in slow relaxation.

Strain-time and stress-time data in the sinusoidal test **(Figure 1F)** were fitted with a sinusoidal function[3,4], and phase shift was calculated:

$$\sigma(t) = A_\sigma \sin(2\pi f t + \varphi_\sigma) + \sigma_0, \quad (3)$$

$$\varepsilon(t) = A_\varepsilon \sin(2\pi f t + \varphi_\varepsilon) + \varepsilon_0, \quad (4)$$

$$\gamma = \varphi_\sigma - \varphi_\varepsilon\,, \quad (5)$$

where $\sigma(t)$ is stress as a function of time, $\varepsilon(t)$ is strain as a function of time, t is time, A_σ and A_ε are amplitudes of stress-time data and strain-time data, respectively, f is oscillation frequency, φ_σ and φ_ε are phase angles of stress-time data and strain-time data, respectively, σ_0 and ε_0 are the constants, and γ is phase shift.

Structural discontinuities due to the damage reduce the effective area that resists stretching in a material and can be represented as $A_{\text{before damage}} = A_{\text{measured}}(1 - D_\sigma)$. By considering that $\sigma = F/A$, the damage parameter D_σ is formulated as follows:

$$\sigma_{\text{before damage}} = \frac{\sigma_{\text{after damage}}}{1-D_\sigma} \quad (6)$$

$$D_\sigma = 1 - \left(\frac{\sigma_{\text{before damage}}}{\sigma_{\text{after damage}}}\right), \quad (7)$$

where $\sigma_{\text{before damage}}$ is maximum stress before damage, and $\sigma_{\text{after damage}}$ is maximum stress after damage, and $0 \leq D_\sigma \leq 1$.[5]

Correspondingly, D_ε explains the damage-dependent effective strain that can be described as $\varepsilon_{\text{before damage}} = \varepsilon_{\text{after damage}}(1 - D_\varepsilon)$, where $\varepsilon_{\text{after damage}}$ and $\varepsilon_{\text{before damage}}$ are strain after and before damage, respectively. Thus, D_ε is formulated as follows:

$$D_\varepsilon = 1 - \frac{\varepsilon_{\text{after damage}}}{\varepsilon_{\text{before damage}}}, \tag{8}$$

where the strain is determined based on 0.2 MPa criteria, and $0 \leq D_\varepsilon \leq 1$.[5,6]

Strain increase (λ_ε) describes the difference between the strain after damage and before damage:

$$\lambda_\varepsilon = \varepsilon_{\text{after damage}} - \varepsilon_{\text{before damage.}} \tag{9}$$

Yielding points ($\varepsilon_{\text{yield}}$ and σ_{yield}) were determined by observing the 0.6% offset from the linear region of the stress-strain curve.[7]

S2) Region of interest (ROI) selections in the manual segmentation

The two samples in **Figure 4A** and **4B** represent a successful clustering method for segmentation. However, in the rest of the samples, clustering was not successful due to some subtle variance in the Ta_2O_5-cNP contrast agent distributions that may be caused by excess unbound nanoparticles. Thus, for statistical quantitative purposes, manual segmentation was performed by selecting a region of interest (ROI) of the IFM and fascicular regions that are close to each other. Some of ROI selections are shown in **Figure S-1**.

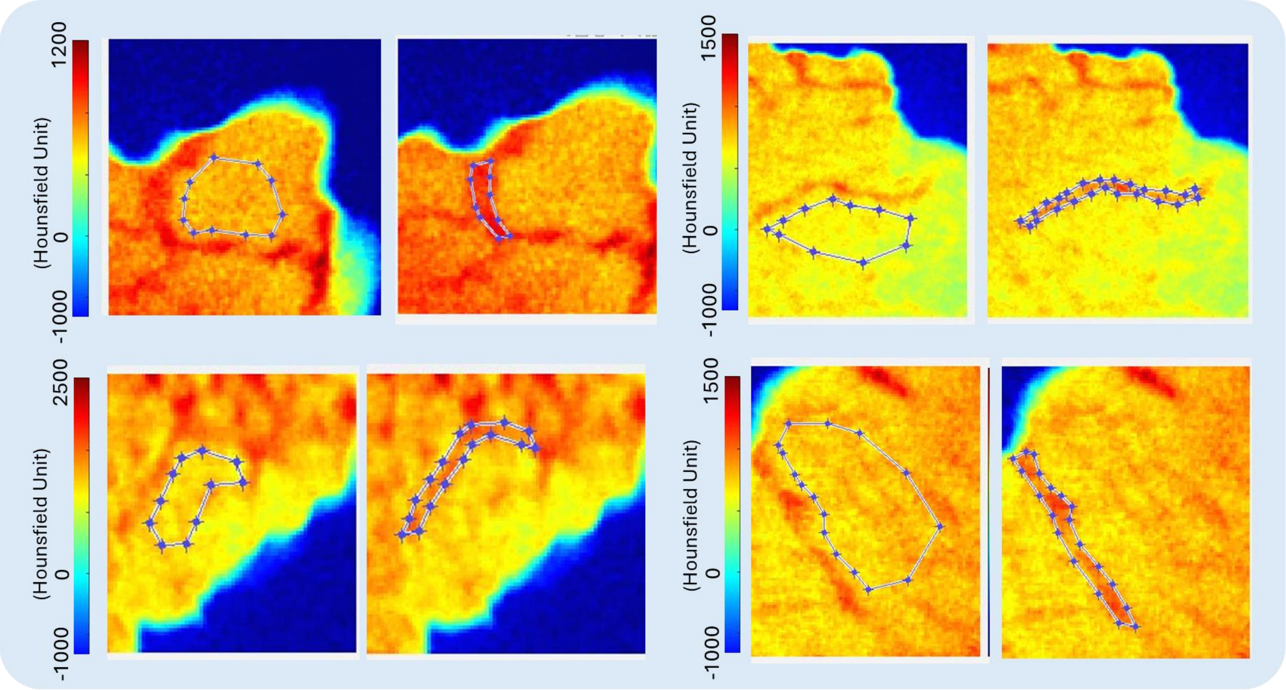

Figure S-1. Four representative transverse-plane samples showing the X-ray attenuation and ROI selection of the fascicular regions (left images of image pairs) and IFM (right images of image pairs). The color scale of each sample was adjusted during analysis for visualization purposes during the segmentation.

S3) Correlations between biomechanics and iodixanol CECT at 8-hour timepoint

In addition to the maximum partition (P_{max}), the correlations between biomechanical properties and iodixanol bulk partition were evaluated at each timepoint. Significant correlations were observed only at the 8 h timepoint (**Figure S-2**).

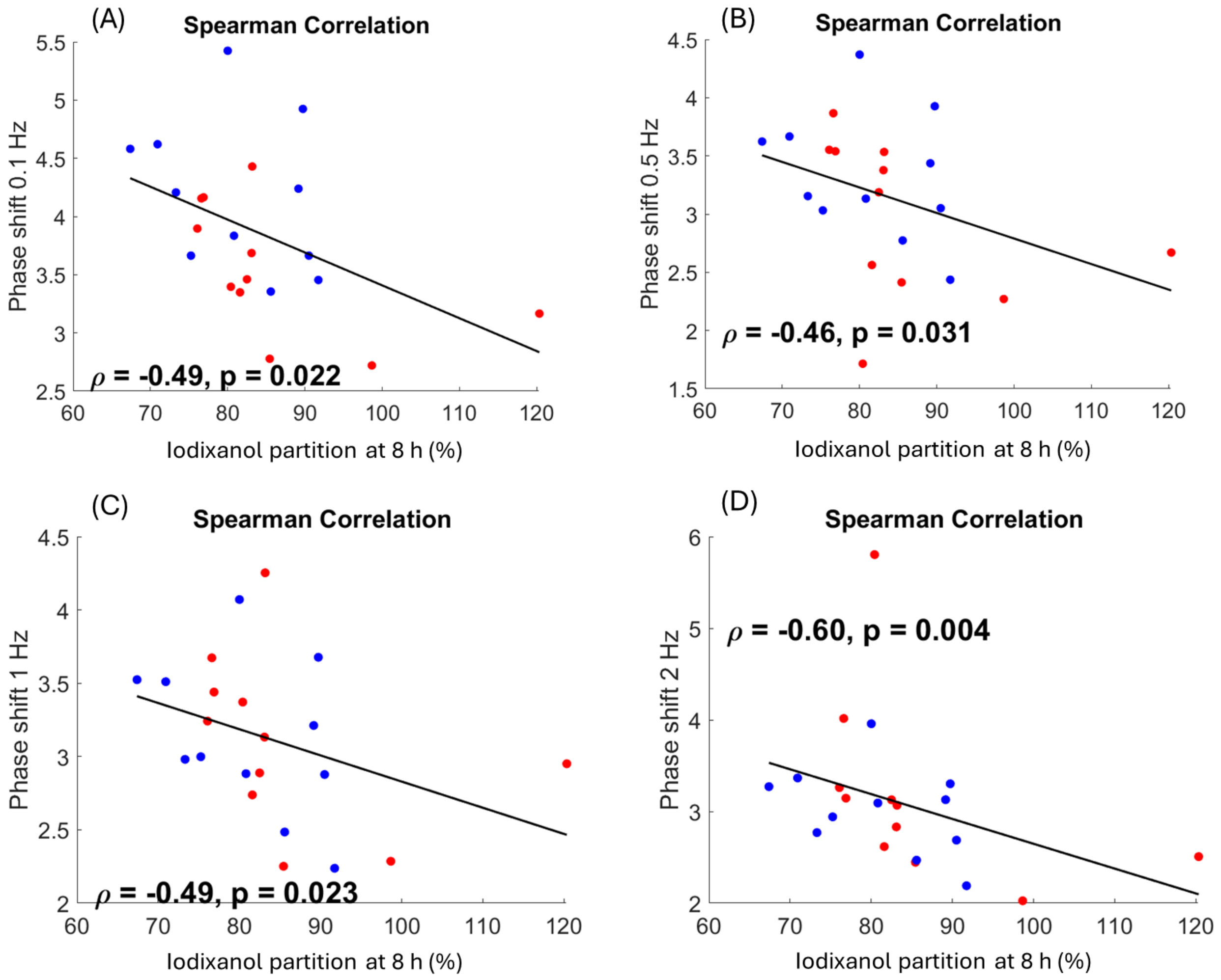

Figure S-2. Spearman correlations between bulk partitions of neutral iodixanol at 8h timepoint with (A) phase shift 0.1 Hz, (B) phase shift 0.5 Hz, (C) phase shift 1 Hz, and (D) phase shift 2 Hz.

References

1. Gheisari A, Ristaniemi A, Haghighatnejad M, et al. 2024. Alterations in mechanical properties of rabbit collateral ligaments eight weeks after anterior cruciate ligament transection. Journal of Biomechanics 176:112350.

2. Gupta HS, Seto J, Krauss S, et al. 2010. In situ multi-level analysis of viscoelastic deformation mechanisms in tendon collagen. Journal of Structural Biology 169(2):183–191.

3. Bonifasi-Lista C, Lakez SP, Small MS, Weiss JA. 2005. Viscoelastic properties of the human medial collateral ligament under longitudinal, transverse and shear loading. Journal Orthopaedic Research 23(1):67–76.

4. Ristaniemi A, Tuppurainen J, Jäntti J, et al. 2024. Comparison of site-specific tensile, compressive, and friction properties of human tibiofemoral joint cartilage and their relationship to degeneration. Journal of Biomechanics 177:112386.

5. Buckley MR, Dunkman AA, Reuther KE, et al. 2013. Validation of an Empirical Damage Model for Aging and in Vivo Injury of the Murine Patellar Tendon. Journal of Biomechanical Engineering 135(4):041005.

6. Duenwald-Kuehl S, Kondratko J, Lakes RS, Vanderby R. 2012. Damage Mechanics of Porcine Flexor Tendon: Mechanical Evaluation and Modeling. Ann Biomed Eng 40(8):1692–1707.

7. Danso EK, Honkanen JTJ, Saarakkala S, Korhonen RK. 2014. Comparison of nonlinear mechanical properties of bovine articular cartilage and meniscus. Journal of Biomechanics 47(1):200–206.
